# Embrained drives to perform extraordinary roles predict schizotypal traits in the general population

**DOI:** 10.1038/npjschz.2016.35

**Published:** 2016-10-12

**Authors:** Ana L Fernandez-Cruz, Ola Mohamed Ali, Gifty Asare, Morgan S Whyte, Ishan Walpola, Julia Segal, J Bruno Debruille

**Affiliations:** 1 Department of Neuroscience, McGill University, Montréal, QC, Canada; 2 Douglas Mental Health University Institute, Montreal, QC, Canada; 3 Department of Psychiatry, McGill University, Montreal, QC, Canada; 4 Department of Psychology, McGill University, Montréal, QC, Canada

## Abstract

Some personal drives correspond to extraordinary social roles. Given that behavioral strategies associated with such drives may conflict with those associated with ordinary roles, they could cause behavioral disorganization. To test whether they do so independent of the factors responsible for full-blown schizotypy and schizophrenia, these drives were assessed in the general population. Two hundred and nine healthy volunteers were individually presented with hundreds of names of social roles in experimental psychology conditions. The task of the participant was to decide whether or not (s)he would consider performing the role at any moment of his/her life. Schizotypal traits were measured with the schizotypal personality questionnaire (SPQ), and delusion-like ideations were assessed by the Peters *et al.* Delusion Inventory. Demographics and social desirability were controlled for. Participants accepting a greater percentage of extraordinary roles had higher SPQ scores. Among the three factors of the SPQ, disorganization was the one best predicted by those percentages. This correlation (*r*=0.40, *P=*7.2E−09) was significantly greater (Fisher Z-transform, *P*=0.003) than the correlation between the percentages of ordinary roles accepted and the SPQ scores (*r*=0.145, *P*=0.044). Reaction times revealed no suboptimal cognitive functioning in high accepters of extraordinary roles and further strengthened the drive hypothesis. Their acceptances of roles were done faster and their rejections took longer than those of low accepters (*P=*5E−12). Culturally embrained drives to do extraordinary roles could thus be an independent factor of the symptoms measured in the normality to schizophrenia continuum.

## Introduction

In recent years, there has been a large number of publications on mirror neurons and on how one imitates others, often subconsciously.^1–3^ These works provide leads to understand how child and adult behaviors are acquired simply and automatically by witnessing others.^1^ Our ability to perform various social roles is probably an example of the complexity of the behaviors that can be learned from seeing others enacting them. The roles performed by family members, friends, and educators are observed and stored for use without conscious effort. Additional sources in our environment, such as television, historical or fictional books, media, and internet, provide us with opportunities to learn about more extraordinary roles and to gain the schemas associated with them. Most importantly, embrained knowledge gained from those sources is associated to a sub- or over-threshold drive to act out those roles. In effect, many children, adolescents, and young adults imitate extraordinary roles, allocating them to each other during live action role-performing games, mimicking superstars at karaoke sessions and super fighters in video games and martial art classes.

The behavioral schemas associated with extraordinary roles often drastically differ from the schemas associated with the ordinary social roles most of us have to enact to get on with our lives. This could be a problem if the drive(s) to perform extraordinary roles is too intense, in other words, if the embrained representations of their schemas is too activated. It may be responsible for a conflict with ordinary roles. Theoretically, such a conflict could be responsible for behavioral disorganization, such as the one measured in the continuum going from normality to schizophrenia via schizotypy.^4^

Our goal was to test this possibility by using a set of names of social roles to see whether the tendency to accept extraordinary ones is associated with schizotypal traits. This association could be stronger for roles opposite to ordinary favorable ones, namely for extraordinary unfavorable roles. In effect, these are the roles whose schemas *a priori* differ the most from the ones associated to ordinary favorable roles. Accordingly, the drive to perform them should be responsible for the greatest conflicts with the roles that have to be performed by most of us on a day-to-day basis. A drive for such roles might thus explain a greater percentage of disorganization variance.

Our aim was to run this test while checking whether the drive to perform extraordinary roles could be a factor of disorganization that differs from other factors found for DSM-diagnosed schizophrenia and schizotypal personality, such as genes and influence of early environment. Our sample was thus taken from the general population as persons with schizophrenia or schizotypy are relatively rare in that population, namely, a little less than 1% for the first^5^ and about 3.7% for the second.^6^ Nevertheless, a continuum probably exists between normality and schizophrenia via schizotypy.^4^ It was thus possible for some of our participants to suffer from a mild version of the neurodevelopmental disorder thought to be responsible for those diagnoses.^7,8^ This would be a problem because this disorder is known to be accompanied by a suboptimal cognitive functioning.^9–13^ Such a functioning could be associated with a greater difficulty at understanding the full meanings of extraordinary roles. It could thus bias our role-acceptance task. To control for such a possibility, reaction times were measured. In effect, reaction times are very sensitive indexes of the difficulty encountered during the cognitive processing of stimuli. In schizophrenia and in high-risk subjects, such reaction times are largely and quasi-systematically longer than in healthy controls.^14,15^

## Results

### Demographics and clinical characteristics

Using a median split, the participants were divided into two subgroups according to the percentages of extraordinary roles they accepted. Table 1 shows the demographics and clinical characteristics of the subjects accepting more extraordinary roles and those accepting fewer. Regression analyses were performed to see whether social desirability scores (SDS) could bias role acceptance. No significant result was obtained. Similarly, the global scores at the schizotypal personality questionnaire (SPQ) were used to divide our participants in a subgroup of high- and a subgroup of low-schizotypy scorers. Table 1 also shows the demographic and clinical characteristics of these two subgroups. The low SPQ one had significantly greater SDS than the high SPQ subgroup (*t* (149)=2.30, *P*=0.023).

### Percentages of role accepted for each condition

**Figure 1:**
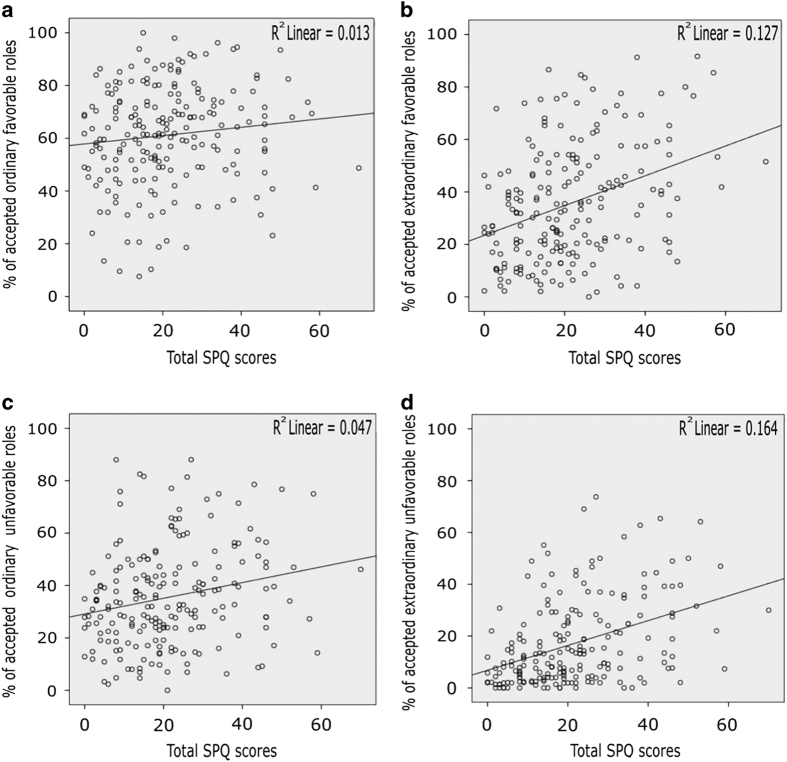
(**a**–**d**) Percentages of roles accepted in each category combination according to SPQ scores. Each small circle represents a participant (*n*=203). Its ‘*x*’ coordinate is his/her SPQ score and the ‘*y*’ coordinate is the percentage of roles (s)he accepted for each category. Scatterplots are for (**a**) ordinary favorable roles, (**b**) extraordinary favorable roles, (**c**) ordinary unfavorable roles, and (**d**) extraordinary unfavorable roles. SPQ, schizotypal personality questionnaire.

The 203 participants (see the 'Materials and Methods' section) responded to 92.8% of the roles. Within these 92.8%, they accepted on average, 49.2% of the ordinary roles, 26.1% of the extraordinary roles, 48.8% of the favorable roles, and 25.8% of the unfavorable roles. Extraordinary roles were the ones whose acceptance percentages most strongly correlated with the total SPQ scores, with the SPQ scores for each of the three factors, with the total Peters *et al.* Delusion Inventory (PDI) scores and with the scores at its subscales (see Table 2 and Figure 1). To check for role specificity, we tested whether the first of these correlations was significantly stronger than that between the acceptances of ordinary roles and the total SPQ scores. This was the case (Fisher Z-transform, *P*=0.003). This effect was also observed for the disorganization factor (Fisher Z-transform, *P*=0.005), the delusion-like-ideation factor (Fisher Z-transform, *P*=0.005), and the interpersonal one (Fisher Z-transform, *P*=0.038). Specificity for extraordinary versus ordinary roles was further demonstrated by the absence of significant difference between the two following correlation coefficients: the one for acceptances of favorable roles and the total SPQ scores, and the one for acceptances of unfavorable roles and the total SPQ scores (Fisher Z-transform, *P*=0.218). No such differences were observed for the total PDI correlation coefficients either. However, the PDI subscales’ (distress, preoccupation, and conviction) correlation coefficients for acceptances of extraordinary roles were significantly greater than those for acceptances of ordinary roles (Fisher Z-transform, *P*=0.040, *P*=0.005, *P*=0.008, respectively).

Looking at the category combinations among extraordinary roles, the unfavorable ones were those whose acceptance percentages most strongly correlated with the total SPQ scores and with the SPQ scores for each of the three factors (see Table 2 and Figure 1). To check for specificity, we tested whether the first of these correlations was significantly stronger than that between acceptances of ordinary favorable roles and total SPQ scores. This was the case (Fisher Z-transform, *P*=0.002). This was also observed for the disorganization factor (Fisher Z-transform, *P*=0.003), the delusion-like-ideation factor (Fisher Z-transform, *P*=0.005) and the interpersonal one (Fisher Z-transform, *P*=0.018). The specificity for extraordinary versus ordinary roles was further demonstrated by the presence of significant differences between the two following correlation coefficients: the one for acceptances of extraordinary unfavorable roles and the total SPQ scores, and the one for acceptances of ordinary unfavorable roles and the total SPQ scores (Fisher Z-transform, *P*=0.014). No such differences were observed for the total PDI correlation coefficients and for the PDI subscales’ correlation coefficients.

Regardless of their favorability, the higher the SPQ score, the greater the number of extraordinary roles that were accepted (see Figures 1b and d). A multiple regression analysis was performed to explore whether percentages of accepted roles for each category combination could complement each other in predicting total SPQ scores and/or whether some conditions would render others insignificant. A significant regression equation was found (F(4, 202)=11.57, *P=*2E−08), with an *R*^2^ of 0.189. The standardized *β*-coefficients were as follows: positive for extraordinary unfavorable (*β*=0.280, *P*=0.026), for extraordinary favorable (*β*=0.238, *P*=0.047), and for ordinary unfavorable roles (*β*=0.071, *P*=0.511), and negative for ordinary favorable roles (*β*=0.212, *P*=0.041). The same regression analysis was performed to predict disorganization. It was significant (F(4, 202)=10.14, *P=*1.7E−7), with an *R*^2^ of 0.170. The standardized *β*-coefficient were as follows, positive for extraordinary unfavorable (*β*=0.259, *P*=0.042), for extraordinary favorable (*β*=0.226, *P*=0.063), and for ordinary unfavorable roles (*β*=0.095, *P*=0.386), and again negative for ordinary favorable roles (*β*=−0.235, *P*=0.026).

**Figure 2:**
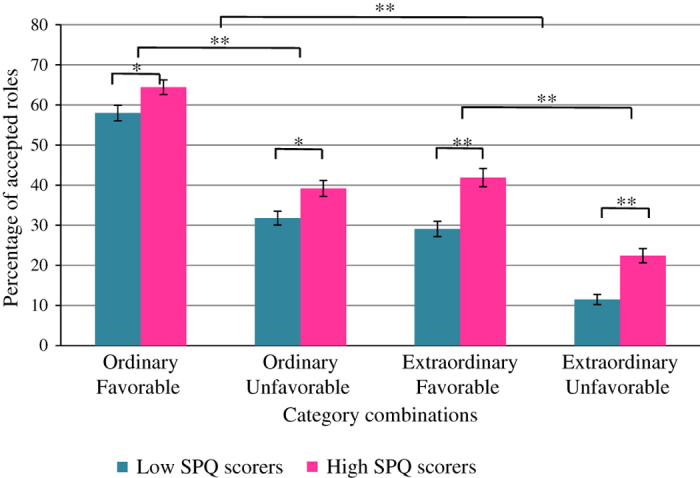
Percentages of social roles accepted in each category for high- and low-SPQ scorers and standard errors (vertical bars) for the 203 participants. *P<0.05, **P<0.0001. SPQ, schizotypal personality questionnaire.

The percentages of social roles accepted in each category combination for each of the two SPQ subject-subgroups are shown in Figure 2. The results of the repeated-measures analysis of variance (ANOVA) revealed that ordinary and favorable roles, independently, were more often accepted than their counterparts (F(1, 201)=435.78, *P*≪0.000001) and (F(1, 201)=582.84, *P*≪0.000001), respectively. Also, there was an ordinariness×SPQ group interaction (F(1, 201)=5.44, *P*=0.021) but no three-way interaction was observed. In general, across all categories, individuals with high SPQ scores accepted more social roles than those with low SPQ scores (F(1, 201)=18.95, *P*=0.000013; see Figure 2).

### Reaction times

**Figure 3:**
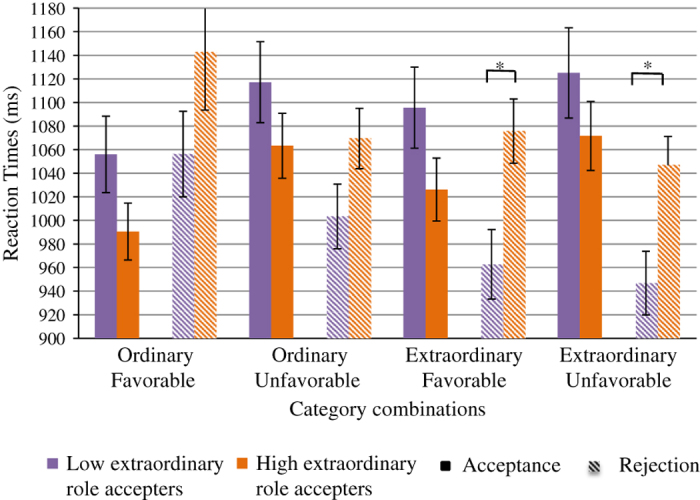
Mean reaction times and standard errors (vertical bars) for accepted (plain rectangles) and rejected (crosshatched rectangles) social roles for high and low extraordinary roles accepters. *P<0.05.

Mean reaction times are shown in Figure 3

High accepters of extraordinary roles appeared faster at accepting all roles (*M*=1038, s.d.=258) and slower at rejecting them (*M*=1,084, s.d.=303) than low accepters (*M*=1,099, s.d.=299) and (*M*=992, s.d.=257), respectively. The mixed-model ANOVA revealed that this acceptance×subgroup interaction was significant (F(1, 163)=55.56, *P*=5E−12). *Post hoc* analyses using independent samples *t*-tests showed that high accepters were significantly slower at rejecting extraordinary favorable and unfavorable roles than low accepters, (*t*(177)=−3.23, *P*=0.001) and (*t*(177)=−3.20, *P*=0.002), respectively.

All the participants reacted faster to ordinary than to extraordinary roles for both acceptances and rejections (F(1, 163)=5.49, *P*=0.020). Globally, accepting roles took longer than rejecting them (F(1, 163)=4.37, *P*=0.038). The mixed-model ANOVA showed absolutely no effect of SPQ scores on the reaction times (F(1, 163)=0.024, *P*=0.876). There was an ordinariness×favorability×SPQ interaction (F(1, 163)=4.12, *P*=0.044), but *post hoc* analyses using independent samples *t*-tests revealed no difference between the participants with high- and those with low-SPQ scores for any combinations.

## Discussion

The drive to perform extraordinary social roles was quantified in healthy subjects to see whether it predicts schizophrenia-like symptoms, particularly subclinical disorganization. As expected, the greater the number of extraordinary roles participants were willing to engage in, the higher their schizotypy scores as measured by the SPQ (Figures 1b and d). The multiple regression revealed that the percentages of acceptances of the roles that differ the most from ordinary favorable roles, that is, the extraordinary unfavorable roles, were the ones that best predicted the clinical scores. This supports the idea that the drive for performing roles that are the most difficult to reconcile with those that most people have to perform in everyday life may engender the subclinical disorganization symptoms measured by the SPQ (i.e., bizarre behavior, thought disorders, unusual thoughts, poor insight, and difficulty in abstract thinking).^16^ In accordance with that view, the multiple regression suggested that the tendency to accept ordinary favorable roles had some protective effect against schizotypal traits. Across all the categories, individuals with higher SPQ scores accepted more social roles than those with lower scores. All these results are reminiscent of the numerous anecdotes, so often published in the media, about the disorganization of famous artists and the extraordinary roles they perform in society, now and in the past.^17^ On the other hand, works, such as the study of Twomey *et al.*,^18^ link psychoticism to openness to experience, sensation-seeking and creativity. Similarly, it was observed that, among writers and actors, scores for psychoticism were higher than among less ‘creative’ individuals^19^ and Andreasen noted in 1996 (ref. 20) that highly creative people had higher scores on measures of psychopathology than less creative people. In our case, this is akin to individuals who accept a lesser percentage of extraordinary social roles having lower SPQ scores.

Further supporting the drive hypothesis, high accepters of extraordinary roles appeared ‘more enthusiast’ at accepting roles and more reluctant at rejecting them as they took less times for acceptances and more times for rejections than low accepters. This could be related to a higher baseline level of activation of the brain representations of these roles in the participants with higher SPQ scores. These results were obtained in a sample of the general population. Given the low frequency of schizotypy and of schizophrenia in that population, it is highly unlikely that those who accepted more extraordinary roles did it as a consequence of some pre-existing schizophrenia factors. One could eliminate an effect of the cognitive deficits found in the continuum going from normality to schizophrenia via schizotypy.^9–13,21^ In the task, these deficits would have induced longer reaction times for those who accepted more extraordinary roles or for the subgroup with higher SPQ scores.^21^ This was not observed. In fact, the absence of reaction time differences has been previously observed in a study looking at schizotypy and the N400 potential.^22^ Thus, the participants who accepted more extraordinary roles did not do it because they were less cognizant of their inappropriateness. Their strategy was similar to that of other participants: all subjects were quicker at accepting ordinary or favorable roles than they were at accepting extraordinary or unfavorable roles (Figure 3).

Future studies should ask the participants to rate the strength of their will to accept each role as this rating might permit to explain more disorganization and schizotypy variance than the acceptance percentages and the reaction times collected here. These studies should also openly ask participants which roles, even extraordinary ones, they would have likely considered. These roles would enrich the list and their strength ratings may further increase the individual fit. Furthermore, the paradigm could be tried in other patient populations suffering from mental disorders that may include disorganization and other psychotic features, such as schizophrenia, bipolar disorder, postpartum psychosis, and schizoaffective disorder. The drive to perform extraordinary roles could exist in any of them. The drive to perform unfavorable roles should also be studied in disorders including a lack of empathy, such as antisocial diagnosis. The general follow-up of patients might be improved by taking their drives into account in the psychotherapy process.

## Materials and methods

### Participants

A set of 209 healthy volunteers was recruited through online advertisements, posted on two sites for the general population (Craigslist and Kijiji) and one site for university students: McGill classifieds. This set included two samples that underwent similar versions of the experiment (see the procedure section). The first sample encompassed 159 participants (97 women) who were between 18 and 30 years of age (*M*=22.80, s.d.=3.19) and had a number of years of education comprised between 10 and 21 (*M*=14.56, s.d.=1.89). Eight of its individuals did not disclose their education level. The other sample involved 44 individuals (25 women) between the ages of 18 and 30 (*M*=22.07, s.d.=2.77) with an education between 12 and 18 years (*M*=14.79, s.d.=1.21). All the participants were native English speakers or had acquired a minimum of 10 years of English education. They reported no previous history of neurological conditions, intellectual deficits, alcohol or drug abuse, and denied taking medication related to a psychiatric disorder during the two previous years. There were no significant demographic, clinical, and behavioral differences between the two samples. The participants were informed about the purpose of the study and signed a consent form approved by the Research Ethics Board of the Douglas Mental Health University Institute. They were debriefed following the experiment and given monetary compensation for their participation. Six subjects were excluded because they responded to less than 50% of the social roles or because they responded in more than 2,500 ms, which suggests that they were not using the same cognitive strategy as the other participants. Moreover, their acceptance percentages were more than two standard deviations above the mean, making them outliers.

### Questionnaires

All the participants filled out a demographics form and the SPQ. Once preliminary analyses revealed significant effects, the SDS and the PDI were added to refine our measurement of delusional ideas and to enable us to control for social desirability. Thus, 158 participants also took the SDS and 151 participants, the PDI.

The SPQ is a 74-item self-rating scale with an internal reliability of 0.90 to 0.92 and a test–retest reliability of 0.82 to 0.83.^23–25^ It is designed for use in the general population to measure the degree of schizotypy of an individual. Three main factors, disorganization, interpersonal, and delusion-like ideation, account for most of the variance.^26–29^ The disorganization score is calculated by adding the totals obtained for the subscales of odd or eccentric behavior. The delusion-like ideation score is computed by adding the totals obtained from the subscales: ideas of reference and odd beliefs or magical thinking. The interpersonal score is computed by adding the totals obtained for the subscales called excessive social anxiety, no close friends, constricted affect, and suspiciousness/paranoid ideation. The global SPQ scores were used to divide our participants in a subgroup of high- and in a subgroup of low-schizotypy scorers, using a median split.

The PDI is a 21-item questionnaire with an internal consistency of 0.52 to 0.94 and a test–retest reliability between 0.78 and 0.81.^30–32^ It assesses delusion-like symptoms of the general population in a more refined manner than does the SPQ. For each particular delusional idea, the participant is required to rank from 1 to 5 the levels of distress, preoccupation, and conviction associated with this idea.

Last, the Marlowe-Crowne Social Desirability Scale^33,34^ is a 33-item true/false questionnaire used to quantify the tendency of participants to respond in a manner that would make them look better to the researcher (e.g., concealing some liked roles) and therefore be more desirable socially. Participants’ scores can be between 0 and 33. The questions are designed in such a way that the majority of the population provides the same answers. In contrast, individuals with an intense will to be socially desirable give unlikely answers that they think make them look best. Such individuals might thus also tend to accept more favorable roles so as to not appear depreciative or disapproving of roles known to be approved by the majority. The SDS scale was used to control for this possibility.

### Stimuli

Before the experiment, 401 names of social roles (see Supplementary Appendix) were rated on nine-point Likert scales by 42 independent young adult evaluators who were first given a definition of the four criteria used. The ‘extraordinariness’ category had to be rated highly for social roles that would usually exceed human physical or mental capabilities. The ‘unfavorability’ category had to be rated highly for disadvantageous or inconvenient roles. The roles were presented in different random orders across these evaluators. Using median ratings, the set of roles was then split into four ensembles, one for each category combination: (1) ordinary favorable, (2) ordinary unfavorable, (3) extraordinary favorable, and (4) extraordinary unfavorable roles. The first of these four ensembles comprised 107 stimuli, including roles such as jogger, piano teacher, social worker, nurse, and swimmer. The second comprised 92 stimuli, including roles such as vandal, pick pocket, homeless person, and drunk driver. The third comprised 97 stimuli, including roles such as astronaut, Zorro, Hercules, and Prophet. The fourth comprised 105 stimuli, including roles such as devil, bandit, vampire, and slave (see Supplementary Appendix). There were no significant differences across these four ensembles between their mean numbers of letters and their mean frequencies of use as computed from Google books Ngram viewer figures. The set of 401 roles was divided into two subsets of roles balanced for the proportion of each of the four ensembles. Most participants (i.e., 148) were presented with one or the other of these subsets in a balanced way for purpose of brevity but others (55) responded to the whole set.

### Procedure

The subjects were seated comfortably in a dimly lit room and had to stare at a computer screen placed 70 cm from their eyes. The roles were randomly presented one at a time, for 500 ms, in black writing on a white background at the center of a computer screen. Each role was immediately followed by a fixation cross that lasted for 500 ms. The participants were asked to decide as quickly and as accurately as possible whether they could consider themselves performing each role at any moment in their life. The answers were provided by pressing a ‘Yes’ or a ‘No’ button with the index and the middle finger, respectively, a correspondence that was counterbalanced across participants. Only responses given between 300 and 2,500 ms after the onset of the presentation of the role were included in the analysis, to exclude the responses of the trial participants that did not pay enough attention to or were too hesitant. E-Prime, for the first subject sample and Psychtoolbox, a MATLAB plugin, for the second sample, were the softwares used for the presentation of stimuli and the monitoring of participants’ responses.

The 44 participants of the second sample had to perform this task for half of the roles they saw. For the other half of the roles, they had to decide whether another person would consider performing the role, as each role was preceded by a pronoun indicating for whom the participant had to make the decision. Only the responses concerning decisions to perform or not perform the roles for themselves were included in the present study.

### Data processing and analyses

Our conditions included not only the four category combinations mentioned above, but also four other groupings, namely, extraordinary, ordinary, favorable, and unfavorable roles. The percentages of the roles accepted in each of the conditions were calculated by dividing the number of accepted roles by the total number of roles that the participant responded to within that particular condition. Partial Pearson’s *r* correlation analyses were then run between these percentages and the global SPQ scores, and between the percentages and the scores for each of the three factors of the SPQ while always controlling for age and number of years of education. The same was done for the total PDI scores and its subscales. A Fisher Z-transform test was used to compare the strength of the correlations obtained and to see whether they would be stronger for extraordinary than for ordinary roles. A mixed-model repeated-measure ANOVA was then run to analyze the mean percentage of accepted roles, mean reaction times, and clinical characteristics after demographics (Table 1) were controlled for. As previously mentioned, the participants were divided into a high- and a low-schizotypy subgroup by a median split of SPQ scores. Mean percentages of accepted roles and mean reaction times for each of the four categories were calculated for the two subgroups. The mixed-model ANOVA had the percentages of acceptance (four levels: one for each of the four category of roles) as the within-subject factor and schizotypy (high versus low) as the between-subject factor. A similar analysis was performed for mean reaction times (instead of percentages,) adding decision (accepted versus rejected) as a second within-subject factor.

## Figures and Tables

**Table 1:** Demographics and clinical characteristics of high- and low-SPQ scorers and of high and low accepters of extraordinary roles

	*High SPQ,* n*=102* *(60 women)* *mean (s.d.)*	*Low SPQ,* n*=101* *(62 women)* *mean (s.d.)*	*High extraordinary role accepters*, n*=102* *(53 women)* *mean (s.d.)*	*Low extraordinary role accepters,* n*=101* *(69 women)* *mean (s.d.)*
Mean age	22.91 (3.26)	22.37 (2.95)	22.93 (3.27)	22.35 (2.94)
Number of years of study	14.74 (1.76)	14.48 (1.77)	14.82 (1.73)	14.39 (1.78)
Mean scores for delusion-like ideation cluster of SPQ	13.68 (5.98)**	4.07 (3.65)	10.76 (7.14)**	7.01 (6.14)
Mean scores for interpersonal cluster of SPQ	13.28 (7.03)**	4.18 (3.44)	10.35 (7.87)*	7.14 (6.04)
Mean scores for disorganization cluster of SPQ	8.21 (3.49)**	3.11 (2.67)	6.91 (4.19)**	4.42 (3.43)
Mean scores for total SPQ	32.01 (10.91)**	10.47 (5.62)	25.60 (14.70)**	16.94 (11.44)
Mean scores for total PDI^a^	22.40 (30.31)*	8.70 (14.93)	20.00 (28.59)	12.77 (21.92)
Mean scores for PDI distress^a^	21.58 (14.01)**	7.71 (6.44)	17.89 (13.96)*	12.19 (11.51)
Mean scores for PDI preoccupation^a^	21.77 (13.15)**	8.43 (6.56)	18.98 (14.01)**	11.86 (9.32)
Mean scores for PDI conviction^a^	25.41 (14.98)**	9.82 (7.92)	22.29 (15.67)**	13.66 (11.45)
Mean scores for total SDS^b^	13.29 (4.12)*	15.06 (5.33)	13.78 (4.63)	14.51 (4.97)

Abbreviations: PDI, Peters *et al.* Delusion Inventory; SDS, social desirability scale; SPQ, schizotypal personality questionnaire.

a *N*=86 for high SPQ/role-acceptance group and *N*=72 for low SPQ/role-acceptance group. Numbers of participants who, in addition to the SPQ, also had the PDI and the SDS questionnaires.

b *N*=80 for high SPQ/role-acceptance group and *N*=71 for low SPQ/role-acceptance group. Numbers of participants who, in addition to the SPQ, also had the PDI and the SDS questionnaires.

*P<0.05,**P<0.0001.

**Table 2:** Pearson’s correlation coefficients between the percentages of acceptance for each social role and the SPQ and PDI scores when controlling for age, education, and SDS

*Clinical scores*	*Ordinary roles*		*Extraordinary roles*		*Favorable roles*		*Unfavorable roles*		*Ordinary favorable roles*		*Ordinary unfavorable roles*		*Extraordinary favorable roles*		*Extraordinary unfavorable roles*	
	r	P	r	P	r	P	r	P	r	P	r	P	r	P	r	P
Total SPQ	0.145	**0.044**	0.401	**7.17E−09**	0.251	**0.0004**	0.324	**4.26E−06**	0.078	0.284	0.194	**0.007**	0.359	**3.06E−07**	0.399	**9.34E−09**
Interpersonal	0.113	0.117	0.287	**0.00005**	0.165	**0.022**	0.256	**0.0003**	0.040	0.584	0.174	**0.016**	0.248	**0.001**	0.297	**0.00003**
Delusion-like ideation	0.123	0.088	0.368	**1.45E−07**	0.256	**0.0003**	0.259	**0.0003**	0.097	0.178	0.127	0.079	0.344	**9.35E−07**	0.347	**7.43E−07**
Disorganization	0.127	0.079	0.370	**1.16E−07**	0.212	**0.003**	0.308	**0.00001**	0.055	0.450	0.186	**0.016**	0.320	**5.74E−06**	0.374	**8.63E−08**
Total PDI	0.060	0.502	0.209	**0.019**	0.145	0.106	0.138	0.124	0.061	0.497	0.069	0.442	0.196	**0.028**	0.184	**0.039**
PDI distress	0.056	0.531	0.324	**0.00002**	0.166	0.063	0.243	**0.006**	−0.016	0.858	0.128	0.155	0.291	**0.001**	0.314	**0.0003**
PDI preoccupation	0.098	0.274	0.402	**3.16E−06**	0.245	**0.006**	0.289	**0.001**	0.024	0.786	0.160	0.074	0.382	**0.00001**	0.364	**0.00003**
PDI conviction	0.100	0.265	0.386	**7.86E−06**	0.246	**0.006**	0.274	**0.002**	0.036	0.693	0.150	0.094	0.371	**0.00002**	0.348	**0.00006**

Abbreviations: PDI, Peters *et al.* Delusion Inventory; SDS, social desirability scale; SPQ, schizotypal personality questionnaire.

*N*=195 for the SPQ and its three factors and *N*=128 for the PDI and its three subscales.

Values in bold are statistically significant *P* values (*P<*0.05).
