# Supplementary material for: Embrained drives to perform extraordinary roles predict schizotypal traits in the general population
Source: NPJ Schizophr. 2016 Oct 12;2:16035–. doi: 10.1038/npjschz.2016.35 (PMC5060951; doi:10.1038/npjschz.2016.35)
Supplement: Supplementary Appendix [file npjschz201635-s1.doc]

**Appendix**

Social Roles and their Characteristics

|  |  | Mean ratings computed from the scores of 42 independent evaluators on a 9-point Likert scale | | | |
| --- | --- | --- | --- | --- | --- |
| Stimulus number | Social Role Name | Arousal | Valence | Ordinariness | Favorability |
| 1 | Jesus | 5.67 | 3.60 | 7.24 | 6.22 |
| 2 | Harry_Potter | 4.40 | 3.23 | 6.73 | 6.83 |
| 3 | knight | 4.82 | 3.89 | 6.00 | 5.87 |
| 4 | Buddha | 6.35 | 3.42 | 6.80 | 7.17 |
| 5 | ghostbuster | 4.88 | 4.98 | 6.53 | 4.95 |
| 6 | samurai | 4.93 | 4.81 | 6.58 | 5.83 |
| 7 | Peter_Pan | 5.35 | 3.34 | 6.97 | 6.70 |
| 8 | Superman | 4.41 | 2.81 | 7.43 | 7.34 |
| 9 | fairy | 4.97 | 3.12 | 6.77 | 6.78 |
| 10 | Hindu_God | 5.74 | 4.43 | 6.97 | 5.82 |
| 11 | prophet | 5.09 | 4.26 | 7.12 | 6.10 |
| 12 | Zeus | 4.84 | 4.06 | 7.26 | 5.86 |
| 13 | Einstein | 4.72 | 2.34 | 8.00 | 7.84 |
| 14 | mindreader | 5.37 | 5.29 | 6.59 | 4.56 |
| 15 | medieval_king | 4.57 | 4.93 | 5.81 | 5.06 |
| 16 | God | 4.96 | 3.90 | 8.22 | 5.89 |
| 17 | mermaid | 4.47 | 3.28 | 7.48 | 5.72 |
| 18 | Joan_of_Arc | 5.09 | 4.35 | 6.82 | 5.97 |
| 19 | angel | 5.30 | 3.14 | 7.45 | 7.23 |
| 20 | elf | 5.56 | 3.55 | 7.20 | 5.96 |
| 21 | Gandhi | 5.52 | 2.35 | 7.08 | 7.45 |
| 22 | Noah | 5.89 | 4.18 | 6.92 | 6.12 |
| 23 | Napoleon | 5.19 | 5.47 | 6.57 | 4.76 |
| 24 | Robin_Hood | 4.63 | 2.76 | 6.34 | 6.61 |
| 25 | Dalai_Llama | 6.25 | 2.71 | 7.01 | 7.07 |
| 26 | Shakespeare | 5.18 | 2.96 | 6.76 | 7.20 |
| 27 | Hercules | 4.25 | 3.42 | 7.21 | 6.87 |
| 28 | Ironman | 4.08 | 3.66 | 6.88 | 6.69 |
| 29 | Santa_Claus | 5.89 | 2.74 | 6.87 | 6.85 |
| 30 | Sigmund_Freud | 5.13 | 4.55 | 6.20 | 5.50 |
| 31 | Batman | 4.04 | 3.63 | 7.38 | 6.96 |
| 32 | Brad_Pitt | 4.95 | 3.76 | 5.28 | 6.16 |
| 33 | Angelina_Jolie | 4.92 | 3.69 | 5.61 | 5.88 |
| 34 | Stephen_Hawking | 5.28 | 3.20 | 6.90 | 7.47 |
| 35 | Madonna | 5.05 | 4.10 | 5.93 | 5.79 |
| 36 | Olympic_athlete | 4.35 | 3.33 | 6.38 | 7.28 |
| 37 | Barack_Obama | 4.62 | 4.10 | 5.73 | 6.60 |
| 38 | Bill_Gates | 5.58 | 3.89 | 6.77 | 6.21 |
| 39 | Hillary_Clinton | 5.12 | 4.36 | 5.39 | 5.83 |
| 40 | Moses | 5.32 | 4.12 | 6.78 | 6.44 |
| 41 | Pokemon_trainer | 5.27 | 3.99 | 6.35 | 5.74 |
| 42 | Oprah_Winfrey | 5.67 | 4.26 | 5.79 | 5.84 |
| 43 | Aladdin | 5.11 | 3.13 | 6.34 | 6.21 |
| 44 | Bono_U2 | 4.97 | 4.21 | 5.58 | 5.89 |
| 45 | Winston_Churchill | 5.49 | 4.37 | 5.98 | 6.18 |
| 46 | Jay-Z | 5.47 | 4.39 | 5.37 | 5.86 |
| 47 | Kate_Middleton | 5.99 | 3.59 | 5.35 | 6.36 |
| 48 | Prince_William | 5.88 | 4.16 | 5.18 | 5.58 |
| 49 | Serena_Williams | 4.99 | 3.81 | 6.30 | 6.34 |
| 50 | Queen_Elizabeth_II | 5.99 | 4.08 | 5.89 | 5.54 |
| 51 | Steve_Jobs | 5.35 | 3.93 | 6.73 | 6.86 |
| 52 | Pierre_Trudeau | 5.60 | 4.46 | 5.94 | 5.67 |
| 53 | Bob_Marley | 5.79 | 2.95 | 6.15 | 6.96 |
| 54 | Spiderman | 4.39 | 3.23 | 7.67 | 7.12 |
| 55 | Che_Guevara | 4.38 | 4.60 | 6.17 | 5.81 |
| 56 | Mark_Zuckerberg | 5.58 | 4.59 | 5.95 | 5.68 |
| 57 | Charles_Darwin | 4.94 | 3.29 | 6.51 | 7.01 |
| 58 | Gulliver | 5.43 | 4.48 | 5.71 | 5.72 |
| 59 | Cinderella | 5.58 | 3.13 | 6.35 | 6.50 |
| 60 | Marilyn_Monroe | 5.00 | 3.46 | 6.06 | 6.22 |
| 61 | Princess_Diana | 5.62 | 3.38 | 5.61 | 6.18 |
| 62 | Nelson_Mandela | 4.73 | 2.44 | 6.68 | 7.55 |
| 63 | Cristiano†Ronaldo | 4.76 | 3.56 | 6.01 | 6.56 |
| 64 | Michael_Phelps | 4.72 | 3.86 | 6.62 | 6.45 |
| 65 | Zorro | 4.99 | 3.97 | 6.65 | 6.26 |
| 66 | Salvador_Dali | 4.73 | 3.61 | 6.32 | 6.46 |
| 67 | Cupid | 5.12 | 3.63 | 6.85 | 6.28 |
| 68 | Bugs_Bunny | 5.14 | 2.75 | 6.16 | 6.75 |
| 69 | Uncle_Sam | 5.75 | 5.35 | 5.18 | 4.66 |
| 70 | wizard | 4.28 | 4.10 | 7.29 | 6.30 |
| 71 | Three_Wise_Men | 5.96 | 4.12 | 5.72 | 5.98 |
| 72 | Pied_Piper | 6.03 | 4.31 | 5.72 | 5.40 |
| 73 | army_general | 5.13 | 5.54 | 5.26 | 4.82 |
| 74 | FBI_agent | 4.41 | 5.11 | 6.03 | 5.56 |
| 75 | sultan | 5.61 | 4.85 | 5.79 | 5.43 |
| 76 | ninja | 4.51 | 4.70 | 6.62 | 5.59 |
| 77 | Native_Indian | 5.81 | 3.88 | 4.77 | 6.25 |
| 78 | tightrope_walker | 4.65 | 4.42 | 6.20 | 5.43 |
| 79 | Pharaoh | 5.14 | 4.91 | 6.85 | 5.30 |
| 80 | satyr | 5.24 | 5.03 | 6.10 | 5.10 |
| 81 | faun | 5.98 | 3.82 | 5.35 | 6.06 |
| 82 | leprechaun | 4.81 | 4.50 | 7.07 | 5.47 |
| 83 | psychic | 4.67 | 5.69 | 5.65 | 4.16 |
| 84 | Alice_in_Wonderland | 5.07 | 3.50 | 6.51 | 6.37 |
| 85 | caveman | 5.14 | 5.57 | 4.91 | 4.26 |
| 86 | Invisible_Man | 4.89 | 4.77 | 7.81 | 5.16 |
| 87 | werewolf | 4.08 | 6.67 | 6.71 | 3.32 |
| 88 | evil_wizard | 4.43 | 7.26 | 7.02 | 2.98 |
| 89 | alien | 4.29 | 6.02 | 7.18 | 4.61 |
| 90 | centaur | 4.93 | 4.50 | 6.88 | 5.54 |
| 91 | Elephant_Man | 4.68 | 5.61 | 6.54 | 4.10 |
| 92 | Frankenstein | 4.43 | 6.18 | 6.86 | 3.95 |
| 93 | Hades | 4.39 | 6.75 | 6.60 | 3.42 |
| 94 | Devil | 4.11 | 8.18 | 6.94 | 2.58 |
| 95 | mummy | 4.60 | 5.49 | 6.40 | 5.04 |
| 96 | hunchback | 5.48 | 6.17 | 5.41 | 4.04 |
| 97 | Captain_Hook | 4.95 | 5.34 | 6.00 | 4.42 |
| 98 | slave | 3.91 | 8.27 | 5.33 | 1.53 |
| 99 | dwarf | 6.17 | 4.55 | 5.69 | 4.76 |
| 100 | ghost | 3.98 | 6.62 | 7.20 | 3.33 |
| 101 | Dr._Jekyll | 5.45 | 5.63 | 6.08 | 4.63 |
| 102 | evil_clown | 3.96 | 7.32 | 5.65 | 2.81 |
| 103 | jester | 4.98 | 4.37 | 5.22 | 5.63 |
| 104 | vampire | 4.07 | 6.80 | 7.48 | 2.92 |
| 105 | Cyclops | 4.59 | 6.39 | 7.00 | 4.18 |
| 106 | The_Joker | 4.12 | 5.90 | 6.62 | 4.13 |
| 107 | Medusa | 4.36 | 6.77 | 6.87 | 3.27 |
| 108 | Hitler | 3.11 | 8.53 | 7.16 | 1.62 |
| 109 | alien_abductee | 3.65 | 6.46 | 8.47 | 3.42 |
| 110 | gladiator | 4.09 | 5.86 | 6.33 | 4.99 |
| 111 | Grim_Reaper | 4.36 | 6.66 | 6.24 | 2.91 |
| 112 | cyborg | 4.98 | 4.99 | 5.90 | 4.90 |
| 113 | zombie | 3.72 | 7.14 | 7.84 | 2.84 |
| 114 | pirate | 4.61 | 6.05 | 5.68 | 3.65 |
| 115 | Darth_Vader | 4.83 | 6.37 | 6.35 | 3.87 |
| 116 | witch | 4.79 | 6.97 | 7.14 | 3.20 |
| 117 | Green_Goblin | 4.47 | 6.26 | 6.72 | 3.71 |
| 118 | leper | 5.03 | 6.52 | 5.66 | 3.62 |
| 119 | Kim_Jong_Il | 4.08 | 6.94 | 5.92 | 3.22 |
| 120 | Joseph_Stalin | 4.10 | 6.88 | 6.35 | 3.48 |
| 121 | Bin_Laden | 4.00 | 8.63 | 6.10 | 1.54 |
| 122 | Muammar_Gaddafi | 4.37 | 6.62 | 5.63 | 3.45 |
| 123 | Hannibal_Lecter | 4.63 | 7.05 | 6.19 | 2.82 |
| 124 | Cruella_DeVil | 4.32 | 6.92 | 5.94 | 3.57 |
| 125 | Sumo_Wrestler | 5.05 | 4.43 | 5.45 | 5.47 |
| 126 | Sword_Swallower | 4.15 | 6.26 | 6.92 | 3.67 |
| 127 | ogre | 4.98 | 6.56 | 7.55 | 3.93 |
| 128 | WWF_Wrestler | 4.23 | 5.33 | 5.54 | 4.45 |
| 129 | muscleman | 4.62 | 4.86 | 4.49 | 5.10 |
| 130 | conjoined_twins | 4.59 | 5.78 | 6.96 | 4.11 |
| 131 | contortionist | 4.81 | 4.71 | 5.40 | 5.41 |
| 132 | fire_eater | 4.17 | 5.75 | 6.61 | 4.55 |
| 133 | genocide_victim | 3.36 | 8.49 | 5.74 | 1.18 |
| 134 | Ted_Bundy | 4.34 | 6.42 | 5.58 | 3.55 |
| 135 | evil_stepmother | 3.81 | 8.29 | 5.17 | 2.13 |
| 136 | seawitch | 4.69 | 6.52 | 7.02 | 3.75 |
| 137 | beekeeper | 5.89 | 4.54 | 5.04 | 5.67 |
| 138 | garbage_man | 6.50 | 5.66 | 2.84 | 5.04 |
| 139 | KKK_member | 3.13 | 7.91 | 6.11 | 2.54 |
| 140 | terrorist | 3.09 | 8.86 | 6.21 | 1.38 |
| 141 | cripple | 5.10 | 7.04 | 4.52 | 3.30 |
| 142 | tattooed_man | 5.21 | 4.90 | 3.77 | 4.40 |
| 143 | weed_smoker | 5.80 | 5.19 | 3.05 | 4.33 |
| 144 | convict | 4.35 | 7.37 | 4.72 | 2.50 |
| 145 | abusive_guard | 3.64 | 7.70 | 4.59 | 3.06 |
| 146 | poor_child | 4.33 | 8.12 | 3.31 | 2.65 |
| 147 | homeless_person | 4.79 | 7.50 | 3.75 | 2.87 |
| 148 | burglar | 4.04 | 7.28 | 4.94 | 2.88 |
| 149 | rioter | 4.32 | 6.21 | 4.38 | 3.70 |
| 150 | rebels | 4.54 | 5.58 | 5.00 | 4.76 |
| 151 | child_soldier | 3.23 | 8.83 | 6.08 | 1.11 |
| 152 | amputee | 4.87 | 6.35 | 5.58 | 3.64 |
| 153 | anorexic | 4.20 | 7.75 | 4.60 | 2.64 |
| 154 | goth | 5.67 | 6.11 | 4.60 | 3.76 |
| 155 | dictator | 4.38 | 7.32 | 5.73 | 2.62 |
| 156 | domestically_abused | 2.71 | 8.96 | 4.46 | 1.52 |
| 157 | computer_nerd | 5.75 | 4.64 | 4.31 | 5.84 |
| 158 | protestor | 4.19 | 5.28 | 4.45 | 5.09 |
| 159 | obese_person | 5.25 | 6.89 | 3.35 | 2.96 |
| 160 | starving_child | 4.07 | 8.33 | 4.85 | 1.54 |
| 161 | pregnant_teen | 4.95 | 6.60 | 4.25 | 2.92 |
| 162 | suicidal_person | 2.98 | 7.60 | 5.28 | 1.65 |
| 163 | carjacker | 4.91 | 7.63 | 4.37 | 2.61 |
| 164 | neo-nazi | 3.59 | 7.98 | 5.77 | 2.06 |
| 165 | abused_man | 4.17 | 7.86 | 4.97 | 2.09 |
| 166 | executioner | 4.23 | 7.49 | 5.70 | 3.18 |
| 167 | gangster | 4.32 | 7.20 | 4.76 | 2.83 |
| 168 | self-immolator | 4.18 | 6.08 | 5.73 | 3.92 |
| 169 | cleaning_man | 6.77 | 4.22 | 3.15 | 5.70 |
| 170 | emo | 5.44 | 6.68 | 4.88 | 3.28 |
| 171 | grieving_person | 5.62 | 7.46 | 3.71 | 3.37 |
| 172 | drunk_driver | 3.52 | 8.44 | 4.13 | 1.56 |
| 173 | bullfighter | 4.12 | 6.23 | 5.76 | 4.13 |
| 174 | burn_victim | 3.64 | 8.47 | 5.23 | 1.75 |
| 175 | Muslim_extremists | 3.62 | 7.44 | 5.80 | 2.55 |
| 176 | pickpocket | 4.50 | 7.65 | 4.29 | 2.72 |
| 177 | punk | 5.20 | 5.61 | 4.07 | 4.38 |
| 178 | abused_child | 3.50 | 8.22 | 5.26 | 2.00 |
| 179 | prostitute | 4.69 | 6.64 | 4.26 | 3.24 |
| 180 | losing_boxer | 4.89 | 6.19 | 4.14 | 3.78 |
| 181 | heroin_user | 4.04 | 8.36 | 4.75 | 1.96 |
| 182 | smoker | 5.82 | 6.92 | 2.80 | 2.77 |
| 183 | shoplifter | 5.01 | 7.13 | 3.70 | 2.95 |
| 184 | alcoholic | 4.73 | 7.45 | 3.91 | 2.86 |
| 185 | attacked_woman | 3.34 | 8.76 | 4.76 | 1.73 |
| 186 | terminal_patient | 4.66 | 7.54 | 4.71 | 2.56 |
| 187 | executed_person | 3.96 | 7.56 | 5.40 | 2.75 |
| 188 | gang_member | 3.94 | 7.60 | 4.12 | 2.60 |
| 189 | domestic_abuser | 2.84 | 8.59 | 4.40 | 1.14 |
| 190 | gambler | 5.40 | 6.33 | 3.39 | 3.23 |
| 191 | transvestite | 4.78 | 4.72 | 5.25 | 4.90 |
| 192 | pubescent_teen | 5.18 | 6.25 | 2.75 | 4.10 |
| 193 | blind | 5.50 | 6.89 | 5.23 | 3.20 |
| 194 | bully | 3.88 | 7.65 | 3.27 | 2.17 |
| 195 | child_worker | 5.18 | 6.61 | 4.42 | 3.34 |
| 196 | murder_victim | 3.33 | 8.48 | 5.64 | 1.79 |
| 197 | vandal | 4.56 | 6.94 | 4.41 | 3.39 |
| 198 | chauffeur | 6.72 | 4.20 | 3.36 | 5.60 |
| 199 | hillbilly | 5.88 | 5.71 | 3.87 | 4.22 |
| 200 | plowman | 6.23 | 4.90 | 3.47 | 5.29 |
| 201 | nomad | 5.82 | 4.57 | 5.04 | 5.16 |
| 202 | window_washer | 6.34 | 5.22 | 2.77 | 5.22 |
| 203 | rapper | 4.57 | 4.48 | 4.85 | 5.41 |
| 204 | showgirl | 4.67 | 4.53 | 4.41 | 4.89 |
| 205 | plumber | 6.41 | 4.54 | 3.62 | 5.75 |
| 206 | old_man | 6.30 | 4.33 | 2.86 | 5.98 |
| 207 | sick_person | 5.56 | 7.74 | 2.62 | 2.99 |
| 208 | snake_charmer | 4.74 | 4.76 | 6.16 | 4.90 |
| 209 | miner | 5.39 | 5.90 | 4.69 | 4.56 |
| 210 | lazy_employee | 6.42 | 6.79 | 2.52 | 3.10 |
| 211 | sunburned_person | 5.11 | 6.70 | 3.46 | 3.57 |
| 212 | shooter | 3.91 | 7.48 | 5.29 | 2.96 |
| 213 | armed_robber | 3.54 | 8.05 | 5.16 | 1.71 |
| 214 | landmine_detector | 4.67 | 5.38 | 5.67 | 5.38 |
| 215 | bandit | 4.58 | 6.66 | 5.01 | 3.09 |
| 216 | butcher | 5.45 | 5.41 | 3.66 | 5.42 |
| 217 | guitar_player | 5.82 | 2.69 | 4.40 | 6.97 |
| 218 | teacher | 5.84 | 3.10 | 4.15 | 6.94 |
| 219 | skateboarder | 5.55 | 4.35 | 4.24 | 5.25 |
| 220 | student | 5.79 | 2.94 | 2.29 | 7.07 |
| 221 | public_speaker | 5.08 | 4.34 | 4.23 | 6.11 |
| 222 | crane_operator | 5.97 | 5.03 | 4.17 | 5.41 |
| 223 | working_out | 4.35 | 3.10 | 3.29 | 7.24 |
| 224 | businessman | 5.92 | 4.49 | 3.42 | 5.60 |
| 225 | doctor | 5.45 | 2.90 | 5.28 | 7.71 |
| 226 | ballet_dancer | 5.86 | 3.21 | 5.40 | 6.60 |
| 227 | shopper | 6.13 | 4.43 | 1.79 | 5.20 |
| 228 | pilot | 5.29 | 3.24 | 5.63 | 6.73 |
| 229 | fireman | 4.59 | 3.47 | 5.57 | 7.42 |
| 230 | chef | 5.25 | 3.25 | 4.48 | 7.20 |
| 231 | barista | 6.15 | 3.51 | 3.79 | 5.58 |
| 232 | librarian | 7.24 | 3.36 | 3.33 | 6.75 |
| 233 | gas_pumper | 7.51 | 5.50 | 2.43 | 4.60 |
| 234 | computer_gamer | 5.86 | 5.11 | 3.84 | 5.12 |
| 235 | astronaut | 4.38 | 2.71 | 6.92 | 7.57 |
| 236 | gardener | 6.66 | 3.18 | 3.02 | 7.09 |
| 237 | model | 5.44 | 3.98 | 4.50 | 5.27 |
| 238 | chess_player | 6.42 | 3.51 | 4.55 | 6.13 |
| 239 | lab_researcher | 5.90 | 4.15 | 4.50 | 6.67 |
| 240 | hairdresser | 6.47 | 3.97 | 3.38 | 5.79 |
| 241 | camper | 6.36 | 3.75 | 3.24 | 6.02 |
| 242 | golfer | 6.59 | 4.15 | 3.52 | 5.81 |
| 243 | bicyclist | 5.84 | 3.02 | 3.83 | 7.06 |
| 244 | veterinarian | 5.79 | 3.29 | 4.63 | 7.05 |
| 245 | dentist | 5.95 | 4.40 | 4.14 | 6.22 |
| 246 | sushi_chef | 5.72 | 3.12 | 4.65 | 6.53 |
| 247 | hockey_player | 4.61 | 3.66 | 4.82 | 6.19 |
| 248 | photographer | 5.95 | 3.19 | 3.85 | 7.00 |
| 249 | goal_keeper | 5.96 | 4.19 | 4.22 | 6.58 |
| 250 | yoga_practitioner | 6.56 | 2.96 | 4.10 | 6.52 |
| 251 | music_listener | 6.43 | 2.19 | 3.01 | 7.32 |
| 252 | toddler | 5.21 | 2.84 | 2.56 | 6.64 |
| 253 | driving | 5.72 | 4.18 | 2.76 | 5.84 |
| 254 | swimmer | 5.63 | 3.42 | 4.35 | 6.43 |
| 255 | celebrator | 5.08 | 3.27 | 3.46 | 6.46 |
| 256 | newly_wed | 4.72 | 3.16 | 3.80 | 6.80 |
| 257 | school_child | 6.08 | 3.35 | 2.70 | 6.63 |
| 258 | in_love | 3.47 | 2.28 | 4.85 | 7.28 |
| 259 | mother | 5.42 | 2.30 | 4.45 | 8.45 |
| 260 | lawyer | 5.59 | 4.58 | 4.96 | 6.12 |
| 261 | newspaper_reader | 6.50 | 3.48 | 2.93 | 6.47 |
| 262 | construction_worker | 6.13 | 4.98 | 3.30 | 5.35 |
| 263 | mountain_climber | 4.38 | 3.44 | 5.65 | 6.24 |
| 264 | judo_wrestler | 5.08 | 4.37 | 5.31 | 5.62 |
| 265 | baker | 6.58 | 2.66 | 3.21 | 6.92 |
| 266 | grandmother | 6.14 | 2.20 | 3.99 | 7.79 |
| 267 | grocery_shopper | 6.41 | 4.19 | 1.76 | 5.91 |
| 268 | computer_engineer | 5.77 | 4.25 | 4.33 | 6.45 |
| 269 | ice_skating | 5.47 | 2.98 | 3.84 | 6.30 |
| 270 | pregnant_woman | 4.89 | 3.69 | 3.61 | 6.38 |
| 271 | motorcyclist | 4.77 | 4.45 | 3.72 | 5.73 |
| 272 | praying_person | 6.34 | 4.07 | 3.60 | 5.65 |
| 273 | professor | 5.34 | 3.49 | 5.02 | 6.84 |
| 274 | football_player | 5.38 | 4.36 | 4.66 | 5.62 |
| 275 | botanist | 6.25 | 3.17 | 4.64 | 6.57 |
| 276 | optometrist | 6.56 | 3.99 | 3.92 | 6.00 |
| 277 | disc_jockey | 4.54 | 3.91 | 4.33 | 6.04 |
| 278 | singer | 5.24 | 3.36 | 5.29 | 6.49 |
| 279 | cheerleader | 4.96 | 4.54 | 4.04 | 5.09 |
| 280 | weightlifter | 5.34 | 4.62 | 4.90 | 5.79 |
| 281 | sailor | 5.51 | 3.67 | 4.91 | 6.28 |
| 282 | bartender | 5.57 | 4.13 | 3.98 | 6.07 |
| 283 | police_officer | 4.84 | 5.21 | 4.01 | 5.71 |
| 284 | soldier | 4.30 | 5.43 | 5.10 | 5.13 |
| 285 | fisherman | 6.37 | 4.17 | 3.84 | 6.16 |
| 286 | trail_biker | 5.26 | 4.34 | 4.45 | 5.61 |
| 287 | canoeist | 6.40 | 3.52 | 4.12 | 6.24 |
| 288 | scientist | 5.41 | 3.81 | 5.61 | 7.05 |
| 289 | marathon_runner | 5.46 | 3.79 | 5.60 | 6.72 |
| 290 | tennis_player | 5.21 | 3.38 | 4.39 | 6.69 |
| 291 | horse_rider | 5.36 | 3.33 | 4.18 | 6.28 |
| 292 | flight_hostess | 5.80 | 3.64 | 3.49 | 6.55 |
| 293 | politician | 4.93 | 5.87 | 4.03 | 4.47 |
| 294 | baseball_player | 5.83 | 4.43 | 4.65 | 5.84 |
| 295 | gymnast | 5.29 | 3.36 | 5.81 | 6.31 |
| 296 | newborn_baby | 5.44 | 2.77 | 4.11 | 7.11 |
| 297 | father | 5.83 | 2.38 | 3.86 | 8.01 |
| 298 | dog_owner | 6.10 | 3.13 | 2.51 | 6.51 |
| 299 | waterpolo_player | 5.68 | 3.58 | 4.42 | 5.81 |
| 300 | lacrosse_player | 5.20 | 4.48 | 4.41 | 5.27 |
| 301 | painter | 6.24 | 3.59 | 3.83 | 6.55 |
| 302 | secretary | 6.71 | 4.20 | 2.85 | 5.85 |
| 303 | trumpeter | 5.95 | 3.53 | 4.35 | 6.05 |
| 304 | club_dancer | 5.03 | 4.39 | 3.37 | 4.87 |
| 305 | security_guard | 5.70 | 4.97 | 3.46 | 5.59 |
| 306 | skier | 5.57 | 3.80 | 4.09 | 5.93 |
| 307 | rower | 5.80 | 4.00 | 4.22 | 5.90 |
| 308 | boxer | 4.60 | 5.03 | 4.79 | 5.12 |
| 309 | bowler | 6.45 | 4.00 | 3.52 | 5.55 |
| 310 | farmer | 6.14 | 3.57 | 4.16 | 6.95 |
| 311 | violinist | 6.20 | 2.69 | 5.03 | 6.85 |
| 312 | basketball_player | 4.97 | 3.89 | 4.83 | 5.56 |
| 313 | pole_vaulter | 5.38 | 4.23 | 5.84 | 5.89 |
| 314 | parachutist | 4.37 | 4.29 | 6.06 | 5.75 |
| 315 | motorcycle_racer | 4.61 | 4.23 | 5.17 | 5.28 |
| 316 | lover | 3.84 | 1.75 | 4.24 | 8.06 |
| 317 | curler | 6.48 | 4.00 | 3.95 | 5.45 |
| 318 | jet_skier | 5.00 | 3.84 | 4.62 | 6.17 |
| 319 | judge | 5.49 | 5.25 | 4.90 | 5.97 |
| 320 | diver | 4.94 | 3.98 | 5.03 | 6.07 |
| 321 | surfer | 4.92 | 3.50 | 4.16 | 6.04 |
| 322 | trophy_winner | 4.42 | 3.65 | 5.09 | 6.21 |
| 323 | rafter | 5.10 | 4.09 | 4.64 | 5.73 |
| 324 | birthday_girl | 5.21 | 2.81 | 3.08 | 6.54 |
| 325 | rock_climber | 4.49 | 3.59 | 5.33 | 5.88 |
| 326 | zookeeper | 5.87 | 3.64 | 4.73 | 6.13 |
| 327 | sports_fan | 5.32 | 4.49 | 2.68 | 5.52 |
| 328 | pharmacist | 6.38 | 3.64 | 3.95 | 6.43 |
| 329 | concert_goer | 5.29 | 3.33 | 3.27 | 6.19 |
| 330 | massage_client | 6.48 | 3.65 | 3.38 | 5.85 |
| 331 | movie_goer | 6.76 | 3.34 | 2.08 | 6.47 |
| 332 | carpenter | 6.35 | 3.81 | 3.68 | 6.29 |
| 333 | restaurant_customer | 6.57 | 3.98 | 2.47 | 5.76 |
| 334 | dancer | 4.79 | 3.18 | 4.68 | 6.99 |
| 335 | nature_lover | 5.90 | 2.78 | 4.06 | 7.57 |
| 336 | piano_teacher | 6.41 | 3.14 | 4.35 | 6.74 |
| 337 | jogger | 5.80 | 3.35 | 3.25 | 6.20 |
| 338 | sun_tanning | 5.72 | 4.98 | 2.98 | 3.95 |
| 339 | piano_student | 6.83 | 3.30 | 4.10 | 6.83 |
| 340 | snowboarder | 5.16 | 4.28 | 4.25 | 6.11 |
| 341 | texter | 6.57 | 4.83 | 2.24 | 5.33 |
| 342 | roller_skater | 5.50 | 3.83 | 3.57 | 5.70 |
| 343 | tree_planter | 5.67 | 3.08 | 4.00 | 6.96 |
| 344 | mechanic | 5.83 | 4.33 | 3.53 | 5.74 |
| 345 | meditator | 6.66 | 3.17 | 4.34 | 6.54 |
| 346 | pub_customer | 5.56 | 4.47 | 2.58 | 5.58 |
| 347 | celebrating_Christmas | 4.07 | 2.88 | 3.43 | 7.15 |
| 348 | office_worker | 6.63 | 5.09 | 2.38 | 5.52 |
| 349 | pool_player | 6.21 | 3.75 | 3.31 | 5.87 |
| 350 | dog_walker | 6.87 | 3.20 | 2.60 | 6.47 |
| 351 | sprinter | 4.90 | 3.37 | 5.46 | 6.60 |
| 352 | surgeon | 5.36 | 3.47 | 5.74 | 7.02 |
| 353 | jeweler | 6.31 | 3.82 | 3.76 | 5.71 |
| 354 | tourist | 5.69 | 4.05 | 3.44 | 5.81 |
| 355 | clown | 5.39 | 5.25 | 4.82 | 4.76 |
| 356 | shepherd | 6.18 | 3.78 | 4.10 | 5.97 |
| 357 | graduate | 5.33 | 2.79 | 4.13 | 7.45 |
| 358 | stockholder | 5.46 | 4.93 | 3.61 | 5.43 |
| 359 | detective | 4.99 | 3.76 | 5.55 | 6.49 |
| 360 | lawn_mower | 6.65 | 4.78 | 3.18 | 5.22 |
| 361 | pilgrim | 5.87 | 4.35 | 4.91 | 6.03 |
| 362 | archer | 5.13 | 3.81 | 5.28 | 5.78 |
| 363 | paramedic | 5.04 | 3.13 | 5.25 | 6.88 |
| 364 | polo_player | 5.95 | 4.13 | 4.62 | 6.05 |
| 365 | TV_watcher | 6.57 | 4.27 | 1.55 | 5.01 |
| 366 | architect | 5.99 | 2.86 | 4.96 | 7.02 |
| 367 | forest_ranger | 5.38 | 3.54 | 4.68 | 6.31 |
| 368 | cowboy | 5.36 | 4.11 | 4.72 | 5.86 |
| 369 | personal_trainer | 5.56 | 3.66 | 3.68 | 6.38 |
| 370 | journalist | 5.23 | 3.73 | 4.18 | 6.20 |
| 371 | hiker | 5.49 | 3.35 | 4.10 | 6.39 |
| 372 | crossing_guard | 6.44 | 4.39 | 3.23 | 6.32 |
| 373 | butterfly_catcher | 6.03 | 4.07 | 4.76 | 5.31 |
| 374 | presenter | 6.27 | 4.19 | 3.98 | 6.07 |
| 375 | chemist | 5.78 | 4.04 | 4.69 | 6.29 |
| 376 | radio_show_host | 5.48 | 3.65 | 4.39 | 6.37 |
| 377 | nun | 6.55 | 4.55 | 4.73 | 5.39 |
| 378 | priest | 6.03 | 5.11 | 5.16 | 4.91 |
| 379 | social_worker | 5.98 | 3.33 | 4.69 | 6.90 |
| 380 | astronomer | 5.30 | 3.37 | 5.58 | 6.78 |
| 381 | aircraft_controller | 5.91 | 3.87 | 4.99 | 6.85 |
| 382 | archeologist | 5.33 | 3.19 | 5.27 | 6.59 |
| 383 | bus_driver | 6.86 | 4.51 | 3.08 | 5.85 |
| 384 | train_conductor | 6.37 | 4.28 | 3.36 | 5.77 |
| 385 | wine_taster | 6.26 | 3.53 | 3.91 | 6.42 |
| 386 | manicurist | 6.41 | 4.77 | 3.31 | 5.33 |
| 387 | Nobel_Peace_Prize | 4.20 | 1.73 | 6.98 | 7.87 |
| 388 | movie_director | 4.69 | 3.05 | 5.67 | 6.85 |
| 389 | traveler | 5.00 | 2.98 | 4.23 | 6.92 |
| 390 | story_teller | 6.28 | 2.76 | 3.88 | 7.23 |
| 391 | nurse | 5.27 | 3.01 | 4.47 | 7.39 |
| 392 | voter | 6.14 | 3.74 | 2.85 | 6.62 |
| 393 | writer | 5.88 | 3.06 | 4.84 | 7.39 |
| 394 | playing_child | 5.70 | 2.14 | 2.83 | 7.54 |
| 395 | lifeguard | 5.57 | 2.71 | 4.49 | 7.53 |
| 396 | griller | 6.08 | 4.38 | 3.58 | 5.41 |
| 397 | body_surfer | 5.56 | 4.15 | 4.53 | 5.31 |
| 398 | blogger | 6.39 | 4.29 | 3.29 | 5.10 |
| 399 | playing_paintball | 3.94 | 4.90 | 3.76 | 5.09 |
| 400 | dinner_host | 6.16 | 3.15 | 3.23 | 6.19 |
| 401 | X-ray_technician | 6.26 | 3.99 | 4.28 | 6.16 |
| 402 | runaway | 4.38 | 6.06 | 3.02 | 3.71 |
| 403 | parachutist | 4.48 | 3.77 | 5.23 | 5.56 |
| 404 | paraglider | 5.15 | 3.50 | 5.06 | 5.71 |
| 405 | cousin | 6.13 | 2.50 | 3.10 | 7.35 |
| 406 | mountain biker | 4.02 | 2.88 | 4.40 | 6.94 |
| 407 | European | 5.73 | 3.19 | 4.17 | 6.35 |
| 408 | cave diver | 5.29 | 4.38 | 5.15 | 5.46 |
| 409 | bank | 5.46 | 7.69 | 4.44 | 1.73 |
| 410 | hero | 4.63 | 2.10 | 6.06 | 8.19 |
| 412 | best friend | 3.44 | 1.58 | 4.46 | 8.23 |
| 413 | ice climber | 4.90 | 3.54 | 4.73 | 6.23 |
| 414 | mentor | 5.33 | 2.42 | 5.00 | 7.54 |
| 415 | boss | 5.40 | 4.44 | 4.08 | 5.77 |
| 416 | queen | 5.27 | 3.17 | 5.52 | 6.67 |
| 417 | sidekick | 4.85 | 4.81 | 4.33 | 4.94 |
| 418 | partner | 4.04 | 1.71 | 4.77 | 8.04 |
| 419 | temptress | 5.69 | 4.88 | 4.58 | 4.19 |
| 420 | sand surfer | 5.13 | 2.77 | 4.04 | 6.25 |
| 421 | sorcerer | 5.90 | 5.44 | 6.19 | 4.21 |
| 422 | masochist | 5.48 | 5.52 | 3.96 | 3.90 |
| 423 | bishop | 6.10 | 4.10 | 4.19 | 6.38 |
| 424 | cleopatra | 5.35 | 3.65 | 6.21 | 5.67 |
| 425 | countess | 6.46 | 4.38 | 4.31 | 5.90 |
| 426 | duke | 6.19 | 4.35 | 4.92 | 5.44 |
| 427 | gentleman | 6.65 | 1.67 | 3.92 | 8.00 |
| 428 | hurricaine chaser | 3.35 | 5.67 | 6.15 | 4.06 |
| 429 | confidante | 5.27 | 2.71 | 4.19 | 6.90 |
| 430 | Ebenezer Scrooge | 5.67 | 5.73 | 4.92 | 4.38 |
| 431 | serial killer | 4.38 | 8.67 | 7.02 | 1.73 |
| 432 | lion tamer | 6.02 | 5.06 | 5.44 | 3.81 |
| 433 | adolescent | 3.90 | 4.04 | 2.94 | 5.65 |
| 434 | F1 pilot | 4.13 | 3.73 | 5.92 | 6.33 |
| 435 | test pilot | 4.75 | 3.42 | 4.48 | 6.31 |
| 436 | Russian roulette | 5.79 | 6.04 | 5.25 | 3.25 |
| 437 | electrician | 6.00 | 3.50 | 4.35 | 6.67 |
| 438 | oil driller | 5.19 | 5.17 | 4.48 | 5.35 |
| 439 | taxi driver | 6.83 | 4.27 | 2.75 | 5.27 |
| 440 | truck driver | 5.31 | 3.71 | 3.08 | 6.63 |
| 441 | bomb squad | 5.25 | 5.92 | 6.46 | 4.31 |
| 442 | excavator | 6.71 | 2.98 | 4.17 | 6.46 |
| 443 | coast guard | 4.81 | 3.27 | 5.46 | 7.15 |
| 444 | gas fitter | 5.85 | 4.04 | 4.04 | 5.85 |
| 445 | armored car guard | 5.23 | 5.31 | 4.48 | 4.35 |
| 446 | smoke jumper | 6.02 | 5.06 | 4.63 | 4.88 |
| 447 | nuclear worker | 5.71 | 6.85 | 5.63 | 3.71 |
| 448 | bounty hunter | 5.40 | 5.06 | 5.21 | 4.48 |
| 449 | war correspondent | 5.27 | 5.19 | 5.38 | 5.54 |
| 450 | iron worker | 4.19 | 4.00 | 2.94 | 6.13 |
| 451 | bush pilot | 6.40 | 4.00 | 4.75 | 5.67 |
| 452 | president | 5.60 | 4.96 | 5.13 | 5.71 |
| 453 | coal miner | 5.58 | 5.08 | 3.46 | 5.21 |
| 454 | crab fisherman | 5.31 | 3.92 | 4.73 | 6.92 |
| 455 | daredevil | 4.17 | 4.50 | 7.19 | 3.85 |
| 456 | suicide assailant | 4.79 | 7.77 | 4.38 | 2.04 |
| 457 | mercenary | 6.40 | 6.21 | 4.85 | 3.88 |
| 458 | repossession agent | 5.54 | 6.00 | 4.25 | 4.94 |
| 459 | stuntman | 4.92 | 5.10 | 6.23 | 4.90 |
| 460 | romantic | 4.13 | 1.58 | 4.21 | 7.98 |
| 461 | martial arts expert | 5.31 | 2.60 | 4.44 | 7.00 |
| 462 | immigrant | 5.85 | 3.21 | 3.79 | 6.69 |
| 463 | refugee | 5.94 | 6.52 | 4.60 | 3.67 |
| 464 | police chief | 5.58 | 4.48 | 4.98 | 5.54 |
| 465 | harpist | 5.88 | 2.58 | 5.15 | 6.96 |
| 466 | lumber jack | 6.06 | 3.69 | 3.94 | 5.92 |
| 467 | abortionist | 4.77 | 6.54 | 4.19 | 3.92 |
| 468 | mad scientist | 5.88 | 6.38 | 5.69 | 4.15 |
| 469 | kidnapper | 4.00 | 8.21 | 5.40 | 2.08 |
| 470 | crown prosecutor | 6.19 | 5.58 | 4.25 | 5.38 |
| 471 | Jack Ripper | 6.06 | 6.63 | 5.56 | 3.38 |
| 472 | euthanasiast | 5.42 | 5.50 | 4.79 | 5.71 |
| 473 | grave digger | 5.27 | 6.00 | 4.42 | 4.65 |
| 474 | speleologist | 5.54 | 4.88 | 4.94 | 5.13 |
| 475 | fish gutter | 6.25 | 5.83 | 3.67 | 4.44 |
| 476 | exterminator | 5.88 | 5.33 | 3.75 | 5.52 |
| 477 | plantation owner | 5.56 | 4.52 | 3.79 | 5.85 |
| 478 | chimney sweeper | 6.75 | 4.15 | 3.27 | 5.71 |
| 479 | roofer | 6.06 | 4.71 | 3.08 | 5.44 |
| 480 | shark tagger | 5.31 | 5.17 | 4.96 | 5.40 |
| 481 | divorced | 5.56 | 6.33 | 3.29 | 3.33 |
| 482 | stepmother | 5.42 | 4.00 | 3.06 | 5.35 |
| 483 | magician | 4.69 | 3.19 | 6.48 | 6.23 |
